# Enhanced long-term potentiation and impaired learning in mice lacking alternative exon 33 of Ca_V_1.2 calcium channel

**DOI:** 10.1038/s41398-021-01683-2

**Published:** 2022-01-10

**Authors:** Sheeja Navakkode, Jing Zhai, Yuk Peng Wong, Guang Li, Tuck Wah Soong

**Affiliations:** 1 Department of Physiology, National University of Singapore, Singapore, Singapore; 2 Lee Kong Chian School of Medicine, Nanyang Technological University, Singapore, Singapore; 3 Healthy Longevity Research Programme, Yong Loo Lin School of Medicine, National University of Singapore, Singapore, Singapore; 4 Present Address: Key Laboratory of Medical Electrophysiology, Ministry of Education, Institute of Cardiovascular Research, Southwest Medical University, Luzhou, Sichuan China

**Keywords:** Diseases, Long-term memory

## Abstract

The CACNA1C (calcium voltage-gated channel subunit alpha 1 C) gene that encodes the Ca_V_1.2 channel is a prominent risk gene for neuropsychiatric and neurodegenerative disorders with cognitive and social impairments like schizophrenia, bipolar disorders, depression and autistic spectrum disorders (ASD). We have shown previously that mice with exon 33 deleted from Ca_V_1.2 channel (Ca_V_1.2-exon 33^−/−^) displayed increased Ca_V_1.2 current density and single channel open probability in cardiomyocytes, and were prone to develop arrhythmia. As Ca^2+^ entry through Ca_V_1.2 channels activates gene transcription in response to synaptic activity, we were intrigued to explore the possible role of Cav1.2_Δ__33_ channels in synaptic plasticity and behaviour. Homozygous deletion of alternative exon 33 resulted in enhanced long-term potentiation (LTP), and lack of long- term depression (LTD), which did not correlate with enhanced learning. Exon 33 deletion also led to a decrease in social dominance, sociability and social novelty. Our findings shed light on the effect of gain-of-function of Ca_V_1.2_Δ__33_ signalling on synaptic plasticity and behaviour and provides evidence for a link between Ca_V_1.2 and distinct cognitive and social behaviours associated with phenotypic features of psychiatric disorders like schizophrenia, bipolar disorder and ASD.

## Introduction

Synaptic plasticity is thought to underlie mechanisms of learning and memory and impairments in plasticity mechanisms are implicated in various neurodegenerative disorders such as Alzheimer’s, Parkinson’s and Huntington’s disease and psychiatric disorders such as ASD, schizophrenia and depression [1–6]. Activity-dependent synaptic plasticity such as LTP and LTD are considered to be the cellular models for learning and memory [7, 8]. Induction of LTP in the CA1 region of the hippocampus requires influx of Ca^2+^, through either post synaptic NMDA- (N-methyl-D-aspartate) receptors [9] or voltage gated long-lasting type Ca^2+^ channels (LTCCs) [10]. Specific patterns of stimulation activate NMDA-receptors or LTCCs differentially at distinct regions resulting in NMDA-receptor dependent or LTCC dependent LTP [10, 11].

The Ca_V_1.2 and Ca_V_1.3 are the most prominent LTCC isoforms expressed in neurons and hippocampal pyramidal neurons express more Ca_V_1.2 than Ca_V_1.3 channels [12–14]. Ca_V_1.2 and Ca_V_1.3 channels also mediate excitability of hippocampal pyramidal neurons by activating calcium activated-potassium channels [15]. The high degree of sequence similarity between Ca_V_1.2 and Ca_V_1.3 channels has resulted in the lack of selectivity of LTCC pharmacological activators and blockers [16]. Therefore, the physiological role of these isoforms has been primarily studied from experiments with genetically modified mice [17].

Knockout mice lacking Ca_V_1.3 showed no change in hippocampal LTP and hippocampus dependent behaviours [18]. In contrast, inactivation of Ca_V_1.2 genes in the hippocampus and prefrontal cortex led to selective loss of NMDA-receptor independent schaffer collateral LTP and hippocampus dependent spatial memory, decreased MAPK (mitogen-activated protein kinase) activity, and cyclic AMP (3’,5’-cyclic adenosine monophosphate) dependent activity in pyramidal neurons of the hippocampus [19]. Lack of Ca_v_1.2 channels in glutamatergic neurons impairs theta burst stimulation (TBS)-induced LTP in the hippocampus [20]. Moosemang et al. showed a critical role of Ca_V_1.2 channels in NMDA-receptor independent synaptic plasticity and spatial memory. A loss of CACNA1C in excitatory glutamatergic neurons of the forebrain displayed social behavioural deficit and impaired learning and memory, suggesting the possibility that it may be related to neuropsychiatric disease symptoms [21]. The clinical significance of the role of Ca_V_1.2 in hippocampus dependent behaviours is strengthened by the fact that, *CACNA1C* is a prominent risk gene for mental and psychiatric diseases with cognitive or social impairments such as depression, autism spectrum disorders, schizophrenia and bipolar diseases [22]. Studies have shown that, knock out of BARP (beta anchoring and regulatory protein), which is known to negatively regulate Ca^2+^ channel activity showed multiple behavioural phenotypes including enhanced learning and social interaction [23].

Conditional deletion of *CACNA1C* in the hippocampus and cortex resulted in the impairment of spatial memory and increased anxiety like behaviour [24, 25]. Genetic loss of Ca_V_1.2 alters plasticity and impairs acquisition of fear learning [26]. Alteration in the signalling cascade from Ca_V_1.2 to gene regulation in the nucleus is an important cause for autistic behaviour in Timothy syndrome, which is a rare disorder caused by CACNA1C gene mutations and characterized by multi-organ system dysfunctions [27]. Genomic mapping studies have revealed that mutations in *CACNA1C* is highly ranked among the genes that are associated with autism [28]. Moreover, it has been reported that the complete set of genes that is implicated in autism is largely involved in synaptic plasticity [29]. The clinical significance of the role of Ca_V_1.2 in hippocampus dependent behaviours is strengthened by the fact that, *CACNA1C* is consistently identified as a prominent risk gene in large-scale genome-wide association studies for mental and psychiatric diseases with cognitive or social impairments such as depression, schizophrenia and bipolar disorders [22]. Meta-analysis of GWAS as well as clinical studies have associated *CACNA1C* risk locus rs1006737 with susceptibility to schizophrenia and bipolar disorders [30, 31]. Another risk locus rs2007044 was also found to have influence on synaptic transmission and working memory in patients [32].

Ca_V_1.2 is involved in the coupling of cell membrane depolarisation to an increase in Ca^2+^ permeability and thereby altering gene transcription and synaptic plasticity [33]. An increased activity of Ca^2+^ channels is known to be implicated in the pathogenesis of various neurodegenerative and neuropsychiatric diseases [34, 35]. We had shown earlier that deletion of exon 33 from Ca_V_1.2 channel in mice resulted in a gain-of-function that included conduction of larger currents and increase in single-channel open probability [36]. A “gain-of-function” by exon 33 has not been studied in human brain, while we have shown earlier that in human cardiomyocytes, Ca_V_1.2_Δ33_ channels exhibit a gain-of function [37]. An RNA binding protein *Rbfox1/2*, which was identified in the intronic sequence surrounding Ca_V_1.2 exon 33 was found to enhance the inclusion of alternative exon 33 of Ca_V_1.2 calcium channels during brain development [38]. Abnormal reduction of *Rbfox1/2 (*A2BP1) was reported in a subset of ASD patients [39] and copy number variation (CNV) analysis disrupting *A2BP1* (or *Rbfox1*) have been reported in patients with autism, schizophrenia, epilepsy and mental retardation [40–44].

Here, we reported that exclusion of exon 33 from Ca_V_1.2 channel (Ca_V_1.2-exon 33^−/−)^ resulted in enhanced late-LTP (L-LTP) and reinforcement of early-LTP (E-LTP) to long-lasting LTP. A lack of LTD was also observed and instead resulted in a potentiation. But the upward shift for synaptic plasticity was not followed by improved hippocampus-dependent behaviours. Evaluations of social phenotypes revealed a deficit in sociability and social novelty. Interestingly, the social dominance test which measures aggression showed that the Exon 33^-/-^ mice were more submissive. Our study points to the role of alternative exon 33 of Ca_V_1.2 channels in learning and memory and social behaviours, and its possible wider implications in cognitive and neuropsychiatric disorders.

## Results

### Enhanced LTP in Exon 33^−/−^ mice

**Fig. 1:**
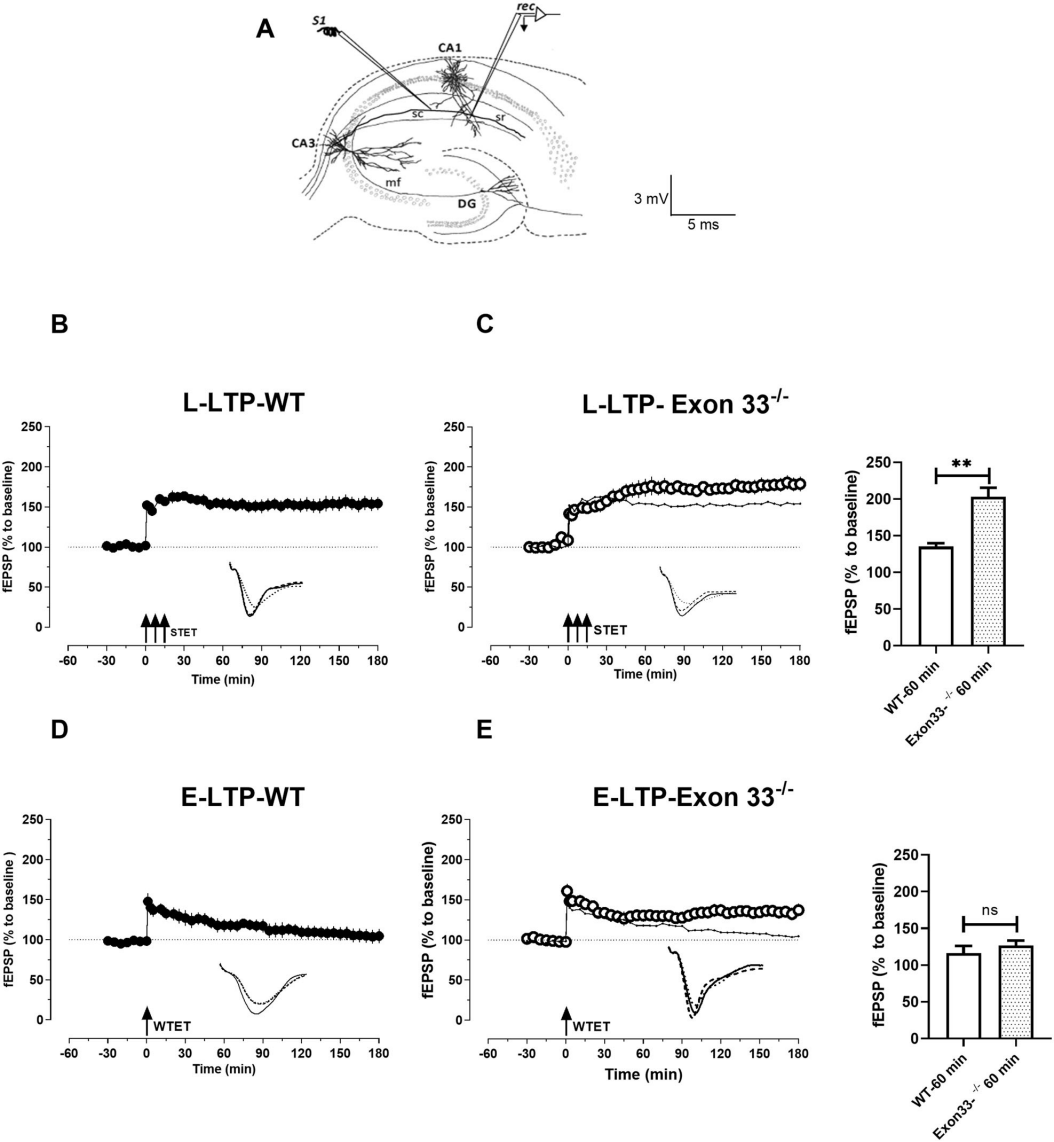
Exon 33^-/-^ mice displayed enhanced LTP and reinforced E-LTP to L-LTP. **A** Schematic representation of a transverse hippocampal slice showing the positioning of electrodes in the CA1 region of hippocampus. One stimulating electrode S1 was placed in the stratum radiatum to stimulate Schaffer collateral fibres and a recording electrode ‘rec’ was placed in the CA1 apical dendritic region. **B** STET in synaptic input S1 resulted in a statistically significant potentiation that maintained for 180 min in WT mice (*P* = > 0.05, *n* = 9). **C** STET in Exon 33^−/−^ mice also resulted in a long-lasting LTP with a higher percentage of potentiation than WT (Fig. 1B) from 30 min and remained stable for 180 min (U-test, 30 min, *P* = 0.002; 60 min, *P* = 0.002; 120 min, *P* = 0.002;180 min, *P* = 0.009, *n* = 9). Dotted line represents Fig B for comparison. **D** WTET in WT resulted only in a short-lasting LTP that decayed to the baseline (Wilcox, 15 min, *P* = 0.03, *n* = 7). **E** WTET in Exon 33^−/−^ mice resulted in a long-lasting LTP that remained significant until 180 min (*P* = > 0.05, *n* = 7). Dotted graph represents **D** for comparison. The dotted line at 100% represents a line for reference. Error bars in all graphs indicate ±SEM. Analog traces represent typical fEPSPs of input S1 recorded 15 min before (dotted line), 30 min after (dashed line), and 180 min (solid line) after tetanisation. Bar graph represents the comparison of potentiation between WT and Exon 33^−/−^ mice at 60 min post tetanisation. Three solid arrows represent the time of induction of L-LTP by STET for the induction of late-LTP. Single arrow represents the time point of induction of E-LTP by WTET. Scale bars: vertical, 2 mV; horizontal, 3 ms. STET-strong tetanisation, WTET-weak tetanisation.

Exclusion of exon 33 from Ca_V_1.2 channels is known to change the electrophysiological properties of the channels, with a gain-of-function effect [45]. Therefore, we were interested to investigate how the gain-of-function of the Ca_V_1.2_Δ__33_ channels might affect neuronal functions like LTP in Exon 33^−/−^ mice. One pathway slice recording method was used to study LTP (Fig. 1A). In order to study L-LTP, a stable baseline was recorded for a minimum of 30 min and a strong tetanus stimulation (STET) was applied to synaptic input S1, which resulted in a long-lasting LTP in WT mice (*n* = 9) (Fig. 1B). A significant potentiation was observed immediately after the first tetanus and it remained stable throughout the recorded time period of 180 min (Wilcox, 1 min, *P* = 0.03; 60 min, *P* = 0.03; 120 min, *P* = 0.03; 180 min *P* = 0.03). We repeated the same experiment in Exon 33^−/−^ mice, which resulted in a more pronounced potentiation that was significant from 1 min until 180 min (Wilcox, 1 min, *P* = 0.03; 60 min, *P* = 0.03; 120 min, *P* = 0.03; 180, min *P* = 0.03) (*n* = 9) (Fig. 1C). Of note, the potentiation in Exon 33^−/−^ mice was significantly higher than the WT mice from 30 min onwards until 180 min (U-test, 30 min, *P* = 0.002; 60 min, *P* = 0.002; 120 min, *P* = 0.002; 180 min, *P* = 0.009). There was no difference in the post tetanic potentiation values of Exon 33^−/−^ mice compared to the WT (U-test, 1 min, *P* = 0.17). This reveals that, Exon 33^−/−^ mice with gain-of-function Ca_V_1.2_Δ__33_ channels resulted in an enhanced potentiation compared to WT mice.

Next, we examined whether short-lasting forms of LTP such as, E-LTP was altered. We applied a weak tetanic stimulation (WTET) to input S1 and detected a decaying form of LTP in WT mice (*n* = 7) (Fig. 1D). LTP was significant only until 15 min (Wilcox, 15 min, *P* = 0.03) and then it decayed to baseline (*P* values at all-time points, *P* > 0.05). However, induction of E-LTP in slices from Exon 33^−/−^ mice, expressed long-lasting LTP (*n* = 7) (Fig. 1E). Application of WTET led to a significant potentiation at 1 min and it remained stable until 180 min (Wilcox, 1 min, *P* = 0.03; 60 min, *P* = 0.03; 120 min, *P* = 0.03; 180 min *P* = 0.03). This shows that the threshold to induce LTP in the Exon 33^−/−^ mice is lower, which facilitate the expression of late-LTP.

### Lack of LTD in Exon 33^−/−^ mice

**Fig. 2:**
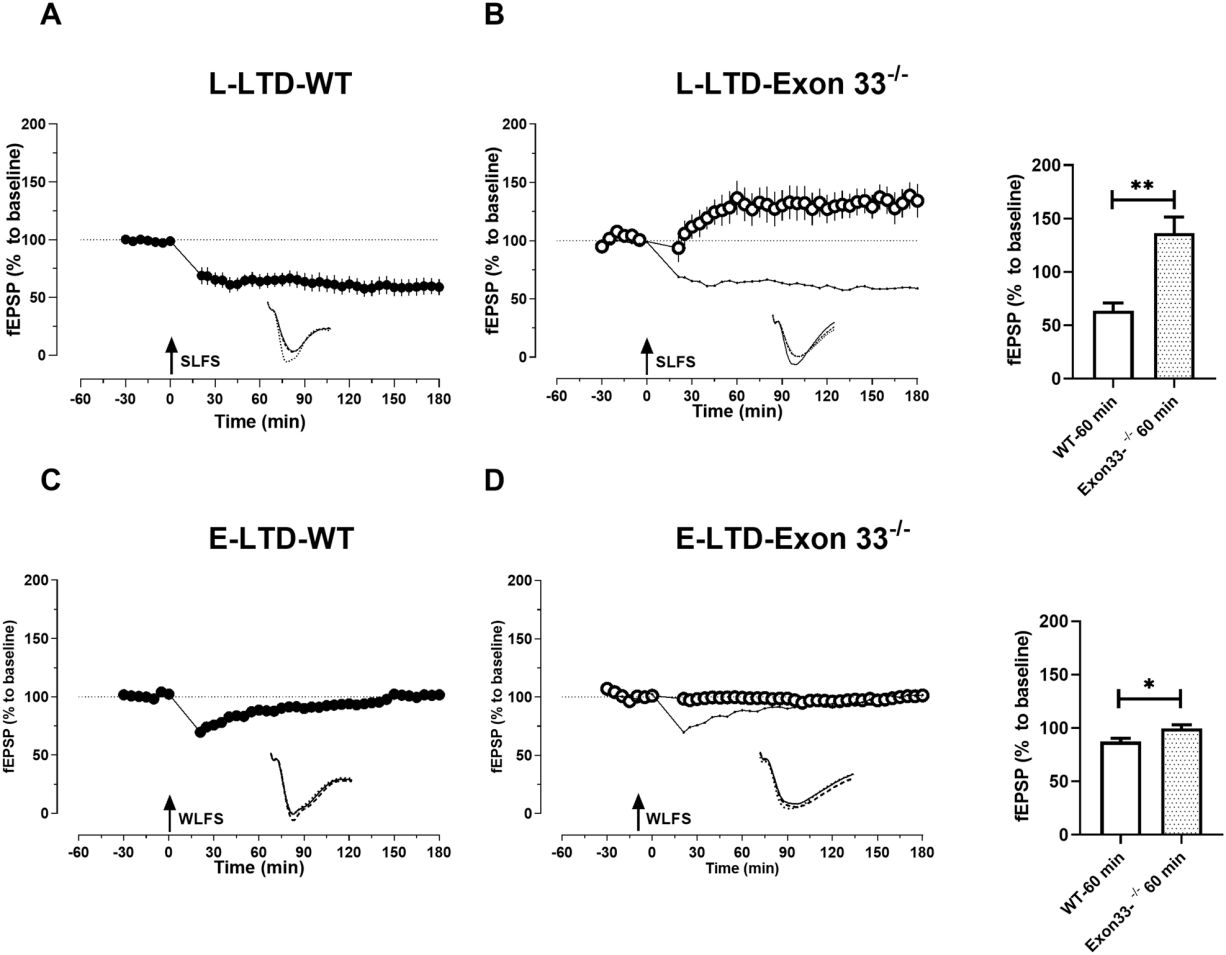
Exon 33^-/-^ mice displayed lack of LTD. **A** Induction of LTD by using SLFS in WT mice, resulted in a stable depression that was statistically significant throughout the recorded time period of 180 min (*P* = > 0.05, *n* = 7). **B** SLFS was delivered to Exon 33^−/−^ mice which resulted in a potentiation after 30 min and was stable for 180 min (*n* = 7). Dotted graph represents **A** for comparison. A significant difference was found between WT and Exon 33^−/−^ mice from 25 min (Utest, 25 min, *P* = 0.004; 60 min, *P* = 0.001; 120 min, *P* = 0.001; 180 min, *P* = 0.001). **C** Induction of E-LTD using WLFS in WT mice showed a depression that was short lasting (85 min, *P* = 0.03, *n* = 8) which then returned to baseline levels. **D** Application of WLFS did not show any significant depression throughout the recording time period of 180 min in Exon 33^-/-^ mice (*P* = < 0.05, *n* = 7). Dotted graph represents **C** for comparison. The bar graphs represents the comparison of potentiation/depression at 60 min time points between WT and to Exon 33^−/−^ mice. Single arrow represents the time point of application of SLFS and WLFS. SLFS- strong low frequency stimulation, WLFS- weak low frequency stimulation. Analog traces and scale bars as in Fig. 1. Error bars in all graphs indicate ±SEM.

LTD is a mechanism for experience induced synaptic weakening and is also a cellular mechanism for learning and memory. As Exon 33^−/−^ mice showed a lower threshold to induce LTP, we were intrigued to determine whether LTD is intact in Exon 33^−/−^ mice. In order to induce L-LTD, a stable baseline was recorded for 30 min and a SLFS was delivered. It resulted in a depression which was significantly lower than the baseline values and remained stable until 180 min in WT mice (Wilcox, 21 min, *P* = 0.03; 60 min, *P* = 0.03; 120 min, *P* = 0.03; 180 min *P* = 0.03) (*n* = 7) (Fig. 2A). When SLFS was delivered to slices from Exon 33^−/−^ mice, we detected the inability of the synapses to undergo depression and instead there was an unexpected slow onset potentiation which stabilised until 180 min (Fig. 2B). SLFS in Exon 33^−/−^ did not induce a depression and the values remained insignificant until 110 min and then from 115 min it showed significant potentiation until 180 min (Wilcox, 115 min, *P* = 0.0.04) (*n* = 7). A significant difference was found between the post SLFS values from 25 min until 180 min when compared between WT and Exon 33^−/−^ mice (U-test, 25 min, *P* = 0.004; 60 min, *P* = 0.001; 120 min, *P* = 0.001; 180 min *P* = 0.001).

In order to evaluate E-LTD, a WLFS was applied to WT slices after recording a stable baseline for 30 min (Fig. 2C). It resulted in a depression and remained significant until 85 min (Wilcox, 21 min, *P* = 0.008; 60 min, *P* = 0.008; 85 min, *P* = 0.03) and then from 90 min onwards it was insignificant compared to its own baseline and remained so until 180 min (Wilcox, 90 min, *P* = 0.08; 120 min, *P* = 0.09; 180 min, *P* = 0.84) (*n* = 8). When WLFS was applied to Exon 33^−/−^ mice no significant depression was observed (Fig. 2D). WLFS did not induce any significant changes in fEPSP values when compared to its own baseline values, from 21 min until 180 min (Wilcox, 21 min, *P* = 0.21; 60 min, *P* = 0.58; 120 min, *P* = > 0.99; 180 min *P* = 0.81) (*n* = 7) (Fig. 2D). When E-LTDs were compared between WT and Exon 33^−/−^ mice, a significant difference was observed in the level of depression from 21 min until 65 min (U-test, 21 min, *P* = 0.0003; 65 min, *P* = 0.02) (Fig. 2C, D).

**Fig. 3:**
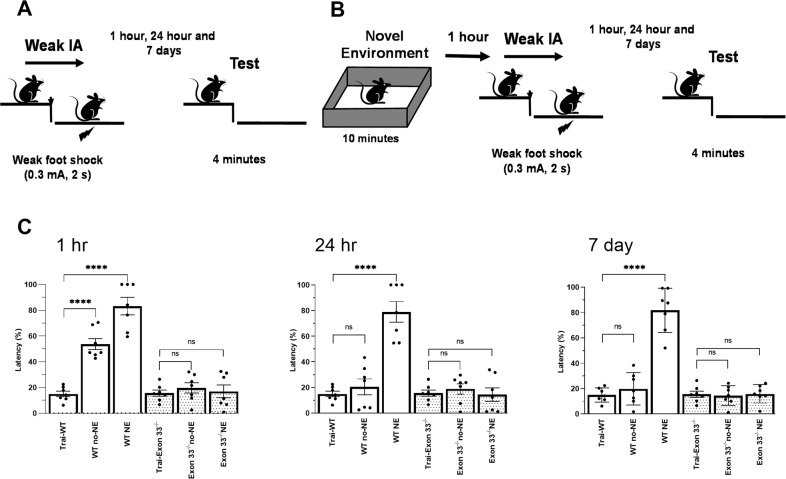
Behavioural tagging is impaired in Exon 33^-/-^ mice. **A** Schematic diagram of experimental protocol used for control experiments in BT. Mice were given weak IA training by giving a weak foot shock consisting of 0.3 mA for 2 s. Step-down latency was tested at 1 h, 24 h, and 7 d post-IA training. The cut-off time for step-down latency was 4 min. **B** Schematic diagram of the experimental protocol used for BT paradigm. Mice were given weak IA training, 1 h after NE (10 min), by providing a weak foot shock. Step-down latency was tested at 1 h, 24 h, and 7 d post-IA. Associative memory was observed only in WT mice (blue bars) exposed to NE. Memory measured 1 h after IA training showed memory retention in WT mice, but not in Exon 33^−/−^ mice (WT no-NE: *P* = < 0.0001; WT NE: *P* = < 0.0001; Exon 33^−/−^ no-NE: *P* = 0.99; Exon 33^−/−^ NE: *P* = > 0.9999 (*n* = 7 for all groups) (**C** 1 h). Memory measured at 24 h shows LTM only in WT mice with NE, but not in WT mice without NE and Exon 33^−/−^ mice (WT no-NE: *P* = 0.97; WT NE: *P* = < 0.0001; Exon 33^−/−^ no-NE: *P* = 0.99; Exon 33^−/−^ NE: *P* = 0.99) (**C** 24 h). Similarly at 7 day, remote memory was seen only in WT mice with NE showing that Exon 33^−/−^ mice was unable to acquire and retain memory (WT no-NE: *P* = > 0.9999; WT NE: *P* = < 0.0001; Exon 33^−/−^ no-NE: *P* = > 0.9999; Exon 33^−/−^ NE: *P* = > 0.9999) (**C** 7 day). Bar graphs representing WT is shown as open bars and Exon 33^−/−^ mice as patterned bars (*n* = 7 from all groups). Error bars indicate ±SEM. Asterisks indicate significant differences between groups (ns = not significant, **p < 0.01, ***p < 0.001, ****p < 0.0001). Error bars indicate ±SEM. Asterisks indicate significant differences between groups. ns represents non-significant (ns) groups.

### Associative memory was impaired in Exon 33^-/-^ mice

As Exon 33^−/−^ mice showed a higher propensity towards potentiation and an inability to induce LTD, we wondered whether associative memory is affected in Exon 33^−/−^ mice. Moncada and Viola have shown earlier that a weak form of memory (mild foot shock) can be consolidated into a strong memory, only if it occurs in close temporal association with a strong stimulation (novel environment (NE)) that induces the synthesis of plasticity proteins [46]. We therefore investigated associative memory using a behavioural tagging (BT) paradigm in WT and Exon 33^−/−^ mice (Fig. 3A, B). We choose the behavioural tagging (BT) paradigm as it shows subtle differences in the behaviour [47]. In the control group (weak inhibitory avoidance (IA) alone), the animals were subjected to a weak foot shock that represents a weaker form of memory and tested for IA memory, by measuring step-down latency at various time points after training (1 h, 24 h, 7 d; Fig. 3A). In the experimental BT group (weak IA with NE), animals were subjected to the same conditions as the control group except that they were first given NE for 10 min prior to the application of a weak foot shock (Fig. 3B). Memory was measured as the latency to step down onto the bars and a longer step-down latency indicates a stronger memory association. Memory was be measured by comparing the step-down latency in the training session to that in the test session (1 h, 24 h, 7 day) (Fig. 3C). The time points are typically used to assess short-term memory (STM: 1 h after the training session), long-term memory (LTM: 24 h after training), and remote LTM (7 days after training) respectively.

When memory was measured at 1 h, WT mice showed significant memory retention in both control and BT group while Exon 33^−/−^ mice did not show any memory for IA training. This shows that STM is intact for WT, while it is impaired in Exon 33^−/−^ mice (WT no-NE: *P* = < 0.0001; WT NE: *P* = < 0.0001; Exon 33^−/−^ no-NE: *P* = 0.99; Exon 33^−/−^ NE: *P* = > 0.9999 (*n* = 7 for all groups) (Fig. 3C 1 h). Comparison of WT with NE and its control without NE shows significant difference at all-time points (WT no-NE vs WT NE; 1 h, *P* = 0.0004; 24 h, *P* = < 0.0001; *P* = < 0.0001).

When LTM was measured at 24 h, WT mice showed significant memory retention only in BT group while Exon 33^−/−^ mice did not show any memory for IA training. This shows that LTM is intact for WT only after NE, while it is impaired in Exon 33^−/−^ mice (WT no-NE: *P* = 0.97; WT NE: *P* = < 0.0001; Exon 33^−/−^ no-NE: *P* = 0.99; Exon 33^−/−^ NE: *P* = 0.99 (*n* = 7 for all groups)) (Fig. 3C 24 h).

Step down latency for remote memories at 7 day show that only WT mice in BT group show memory retention, while all other groups did not show any remote memory (WT no-NE: *P* = > 0.9999; WT NE: *P* = < 0.0001; Exon 33^−/−^ no-NE: *P* = > 0.9999; Exon 33^−/−^ NE: *P* = > 0.9999 (*n* = 7 for all groups)) (Fig. 3C 7 day). Our results show that, Exon 33^−/−^ mice failed to acquire memory for IA learning.

Statistical comparisons between WT and Exon 33^-/-^ mice showed significant difference at 1 h (WT noNE vs Exon 33^-/-^ noNE: *P* = < 0.0001, WT NE vs Exon 33^-/-^ NE: *P* = < 0.0001). Significant difference were observed only in the NE group in both 24 h (WT noNE vs Exon 33^-/-^ noNE: *P* = 0.9, WT NE vs Exon 33^-/-^ NE: *P* = < 0.0001) and 7 day (WT noNE vs Exon 33^-/-^ noNE: *P* = 0.9, WT NE vs Exon 33^-/-^ NE: *P* = < 0.0001).

### Altered social dominance behaviour in Exon 33^−/−^ mice

**Fig. 4:**
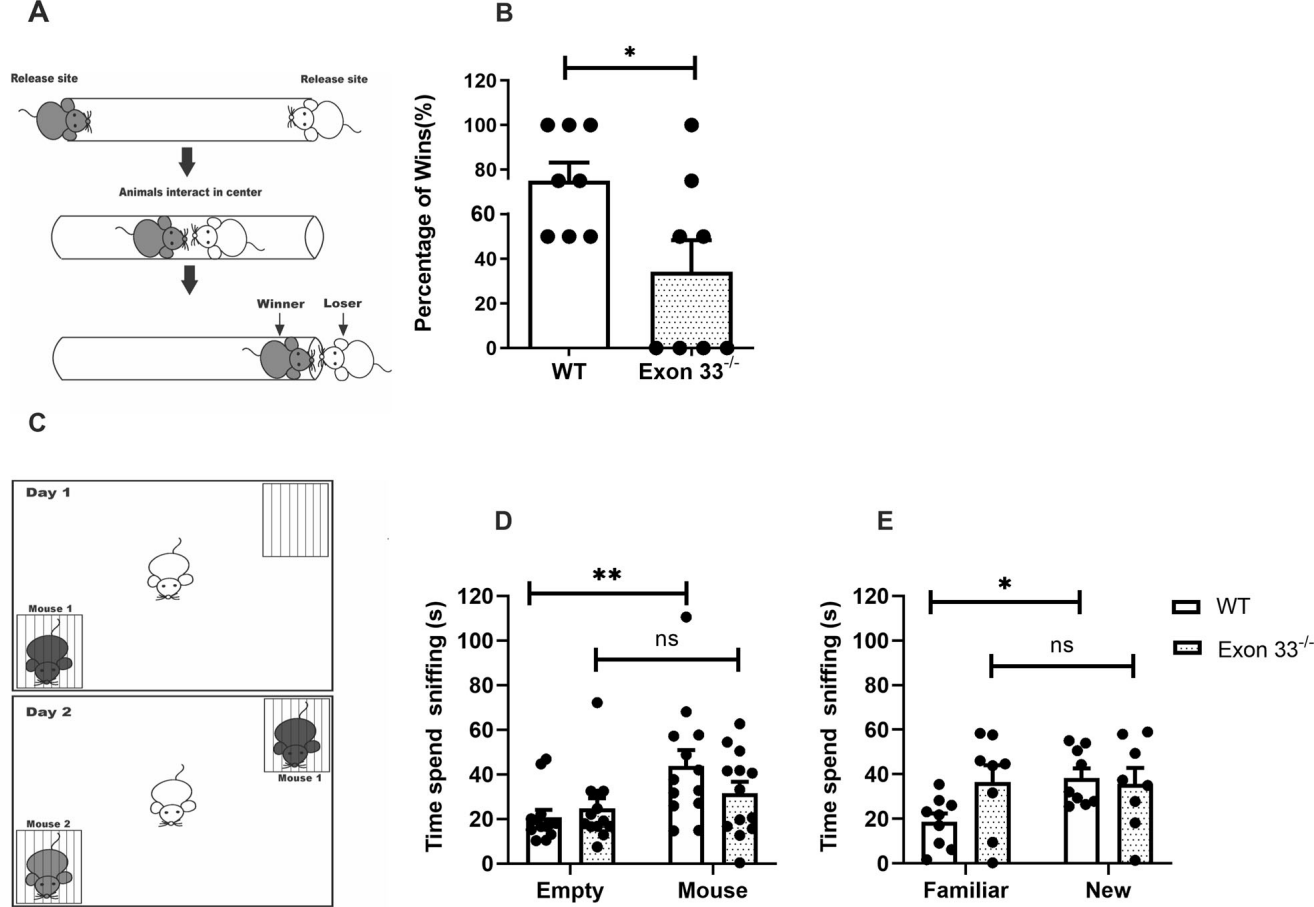
Exon33^-/-^ mice displayed deficits in social dominance, sociability and social preference. **A** Schematic diagram of Tube Dominance Test. Two mice were placed on either side of a clear Plexiglass tube separated by removable separators. As they enter the tube from the release sites at opposite ends, they will interact at the middle of the tube. One of the mice must retreat for the other mice to continue. The dominant mice (grey) shows greater aggression and force the subordinate mice (white) to retreat. The test ends when one mouse pushed the other out of the tube. Winning was scored as binary win lose. **B** Exon 33^−/−^ mice had significantly less wins than WT when tested against WT mice. Student *t*-test was used for statistical analysis (WT *n* = 8, Exon 33^-/-^ *n* = 8, *p* = 0.026). **C** Schematic diagram of social interaction test. The subject mice (white) will be allowed to freely interact with two grid cups in the open field box. On day 1, one empty grid cup and one with Mouse 1 (dark grey) was placed in the box. On day 2, one grid cup will be with the familiar Mouse 1 (dark grey), which has been introduced on day 1 and the other grid cup with a new Mouse 2 (light grey). **D** on day 1, WT mice spent more time sniffing at the grid cup with mouse, while Exon 33^−/−^ mice spent a similar amount of time at both grid cups. Two-way ANOVA with multiple comparisons was used for statistical analysis (WT *n* = 13, Exon 33^-/-^ *n* = 13, F (1, 48) = 8.064, WT *p* = 0.0065). **E** On day 2, when a new Mouse 2 was introduced in the grid cup, WT mice spent more time sniffing at the grid cup with Mouse 2 (new) than Mouse 1 (familiar), while Exon 33 − /− mice still spent an equal amount of time at the two grid cups. Two-way ANOVA with multiple comparisons was used for statistical analysis (WT *n* = 9, Exon 33^-/-^ *n* = 13, F (1, 30) = 2.757, WT *p* = 0.035).

In order to test social dominance, the tube test was done with WT and Exon 33^-/-^ mice placed at the two ends of a clear and narrow tube **(**Fig. 4A**)**. Each Exon 33^−/−^ mouse were tested against every WT mouse, and vice versa, and the number of wins were recorded. Exon 33^−/−^ mice had only 20% wins as compared to 80% wins by WT mice (WT *n* = 8, Exon 33^-/-^ *n* = 8, *p* = 0.026) **(**Fig. 4B**)**. This data suggests that Exon 33^−/−^ mice are less aggressive and less dominant as compared to WT mice.

### Sociability and social novelty changes in Exon 33^-/-^ mice

The social interaction test was used to assess sociability and social novelty in WT and Exon 33^−/−^ mice (Fig. 4C). On day 1 of the sociability test, subject mice encountered a never-before-met Mouse 1 placed in one grid cup and the second grid cup was left empty. Our results showed that on day 1, WT mice spent more time sniffing the grid cup with Mouse 1, while Exon 33^−/−^ mice spent almost the same amount of time sniffing both cups (WT *n* = 13, Exon 33^-/-^ *n* = 13, F (1, 48) = 8.064, WT *p* = 0.0065) (Fig. 4D). This result shows that WT mice prefer to interact with the stranger mouse, while Exon 33^−/−^ mice did not show any preference to stranger mice, suggesting Exon 33^−/−^ mice have lower levels of sociability as compared to WT mice.

On day 2, a second never-before-met intruder, Mouse 2 was introduced to one of the grid cups, with Mouse 1 in the other cup (Fig. 4C bottom). WT mice spent significantly more time sniffing the cup with the intruder mouse 2, while Exon 33^−/−^ mice spent equal amount of time sniffing the two cups suggesting a lack of the preference for the novel Mouse 2 (WT *n* = 9, Exon 33^-/-^ *n* = 13, F (1, 30) = 2.757, WT *p* = 0.035) (Fig. 4E).

## Discussion

We observed that, in Exon 33^−/−^ mice, compared to WT mice, synaptic modification threshold is shifted to favour LTP, as the threshold to induce LTP is reduced in Exon 33^−/−^ mice. This could be attributed to enhanced Ca^2+^ release through the gain-of-function of Ca_V_1.2 Δ_33_ channels, as we had shown earlier that in cardiomyocytes, exclusion of exon 33 resulted in larger current density due to increased open probability and shift of voltage dependent activation in the hyperpolarizing direction [45, 48]. As exon 33 is located in the extracellular linker in domain IVS3-S4 region, it would not be affected by cell-type specific phosphorylation or protein-protein interactions.

The magnitude of Ca^2+^ increase is an important variable that determines the duration of synaptic enhancement [49]. The enhanced Ca^2+^ release might trigger the activation of several kinases like calcium/calmodulin-regulated protein kinases, CaMKII and CaMKIV [50], the cAMP-dependent protein kinase A (PKA) [51], PKC [52] and MAPK/ERKs, that are critical for LTP. The propensity for potentiation with low frequency stimulation that induces LTD, might be due to selective activation of CaMKII instead of calcineurin. CaMKII links Ca^2+^ elevations to downstream cellular targets responsible for LTP [53]. Implication of CaMKII in our study is supported by the finding that excessive Cav1.2 activity, Ca^2+^ entry and CaMKII activity are critical events in the pathology of cardiac arrhythmias [54], which are characteristic of Exon 33^−/−^ mice [36]. In addition, hyperactivation of Cav1.2 by CAMKII is implicated in Timothy Syndrome which includes both social and cardiac symptoms [55].

This propensity towards potentiation was observed in E-LTP as well, since weak tetanus that leads to an early form of LTP resulted in L-LTP, thus strengthening the view that threshold to induce LTP is lower in Exon 33^−/−^ mice. In mice with PSD-95 mutation, L-LTP reached enhanced levels during the first 30 min after high frequency stimulation, when post synaptic kinases and phosphatases are known to regulate plasticity [56, 57], and therefore might be a pathway that is downstream of NMDA-receptors. Unlike these, our results revealed, significant enhanced potentiation after 30 min, and therefore it might be an NMDA-receptor-independent mechanism triggered by enhanced Ca^2+^ entry via Ca_v_1.2_Δ__33_ channels [58]. This view is strengthened by our experiments showing that the L-LTP is not impaired by NMDA-receptor antagonist, AP-5, in Exon 33^−/−^ mice although enhanced potentiation was not observed after AP-5 treatment (Supplementary Fig. 1). This can be explained by the fact that Ca_V_1.2 is also required for NMDA-receptor-dependent LTP in the hippocampus, as loss of Ca_V_1.2 from glutamatergic neurons impairs TBS-LTP, which is NMDA- and local protein synthesis dependent [20]. Thus a gain-of-function of this channel could also affect the NMDA-receptor function, which could account for the lowering of the extra potentiation in Exon 33^−/−^ mice with AP5.

We also observed an inability to induce LTD in Exon 33^−/−^ mice and a propensity towards potentiation in slices where SLFS was delivered to induce L-LTD. This could possibly be due to the increased Ca^2+^ concentrations during SLFS in Exon 33^−/−^ mice compared to WT, which leads to enhanced kinase activation over phosphatases. As Ca^2+^ levels predicts the direction of plasticity, this gain- of-function of Ca_V_1.2_Δ__33_ channels in Exon 33^−/−^ mice might account for the lack of bidirectional plasticity. This shows that enhanced Ca^2+^ influx by Ca_v_1.2 exon 33 deletion induces a metaplastic shift and modification in the threshold to induce different plasticity forms.

Bidirectionally modifiable synapses have more storage capacity, reduced error probability and are capable of limiting the number and strength of potentiated and depressed synapses to an optimum level to facilitate memory storage. As such, the information storage capability of a neural network with only LTP would be far lower than a network which can induce both LTP and LTD. Tonegawa and colleagues have also shown that behavioural and synaptic alterations correlate with a diminished LTD resulting in a shift in the bidirectional plasticity towards potentiation [59]. Similar kind of shifts in metaplasticity or the sliding modification threshold had been seen in GluN2B overexpressing mice [60] and in mice lacking calcineurin, which is critical for LTD [59]. Panx1 channel blockade, like Exon 33 mutants, was found to enhance LTP and causes a shift from LTD to LTP [61]. It was shown that in PSD-95 knockout mice, LTP is enhanced at a wider range of stimulation frequencies and there was an impairment of LTD, followed by impairment of hippocampus dependent spatial memory [58].

As the ability of synapses to undergo bidirectional plasticity is considered as a cellular mechanism for information storage, the shift in the LTP/LTD modification threshold could affect behaviour. Although LTP and LTD are believed to be necessary for modifications that underlie memory formation, numerous studies have shown an association between enhanced LTP and impaired learning. Knockouts of PSD-95, Fmr2, PDE4 and LIMK-1 shows enhanced LTP and impaired learning [58, 62–66]. In line with these inverse correlations, Exon 33^−/−^ mice also showed impaired learning along with enhanced LTP.

In our studies inability to acquire and associate memories might be due to lack of relevant plasticity related proteins that are important for the conversion of shorter forms of memory to long-lasting. It could also be due to inability to set a synaptic tag as the Exon 33^−/−^ mice did not had STM, and therefore was not able to acquire the IA memory. In addition, due to the inability of the synapses to undergo bidirectional plasticity a learning task might activate many synapses causing them to become strongly potentiated and since the synapses cannot be depressed, it might result in the inability to store and retrieve the memory trace. Another reason for the altered plasticity and behaviour might be due to a shift in the source of Ca^2+^ ions. While the mechanism by which bidirectional regulation is carried out is not completely known, the ubiquity of Ca^2+^ as a signalling molecule implies that, any alteration in its function could give rise to a variety of cellular dysfunctions.

Growing evidence has shown the association between LTCC genes and neuropsychiatric disorders including Schizophrenia and autism spectrum disorder, which involves social deficits [67, 68]. Moreover animal models with genetic variation in CACNA1C has revealed potential alterations in social interactions and increased anxiety along with cognitive disabilities [21, 69, 70]. Therefore we were intrigued to study their social behaviour. Mutant mice showed impairments of social behaviours like social dominance showing that they are less aggressive. As Ca_v_1.2 channels are highly expressed in the hippocampus and amygdala, a higher Ca^2+^ concentration in the amygdala could contribute to decreased social dominance in Exon 33^-/-^ mice. The Exon 33^-/-^ mice also did not show any preference to the stranger mouse over the empty grid cup. A normal mouse prefers to spend more time with another mouse than the empty cup and this lack of preference for socialising indicates a social withdrawal behaviour, which is a characteristic of autism. Interestingly Exon 33^-/-^ mice also showed no preferences towards the familiar and intruder mice indicating the lack of preference to novelty. The reduced sociability along with reduced preference for novelty are indicative of an autistic character. Our view is strengthened by the findings from Bader and colleagues, where they showed that heterozygous TS2 (Timothy syndrome 2) neo mice with L-type channel over activity showed multiple behavioural abnormalities which are characteristic of autism spectrum disorders [71, 72]. CACNA1C^+/-^ deletion also leads to increased susceptibility to stress [21]. As social behaviours are influenced by many behavioural characteristics like stress, anxiety, depression, we will study these factors in our future research. As the Exon 33^-/-^ mice showed cardiac symptoms, we also studied general hearing, vision and locomotor activity of the Exon 33^-/-^ mice and they did not show any difference compared to WT mice, thus excluding the possibility of interference with behavioural paradigms (Supplementary Fig. 2).

Most of the gain-of-function mutations in Ca^2+^ channels are linked to psychiatric disorders with cognitive and social disorders like schizophrenia, bipolar disorder, depression and ASDs. Although the mechanisms contributing to social deficits in Exon 33^-/-^ mice is not known, an impairment of CA2 plasticity could underlie the social memory deficits observed as social novelty recognition was impaired in these mice. The mechanisms contributing to social dysfunctions in these mice needs further investigation, but it points to a major role of Ca_v_1.2 in psychiatric disorders.

## Materials and methods

### Animals

Ca_V_1.2-exon 33^−/−^ mice (Exon 33^−/−^ mice) and Ca_V_1.2-exon 33^+/+^ mice (WT mice) were used for the study. Construction of the vector, generation of Exon 33^−/−^ mice and determination of genotypes are as explained in our previous paper [36]. A total of 122 animals were used for this study, which includes 30 mice for electrophysiology, 50 mice for Behavioural tagging (BT), 16 mice for social dominance and 26 mice for social interaction studies. A total of 31 slices were used from 15 WT mice and 29 slices from 15 Exon 33^−/−^ mice for electrophysiology experiments. All electrophysiology experiments were performed using acute hippocampal slices, which were prepared from 7 weeks old male Exon 33^−/−^ and WT mice. BT was also done in 7 weeks old mice, while social dominance and social interaction tests were performed on 16 weeks old mice. All animals were kept on a 12 h light/dark cycle and food and water were provided *ad libitum*. 2-5 animals were housed together. The experimenters were not blinded to the genotype, as the outcome of the studies was not predictable. All experimental procedures were approved by the Institutional Animal Care and Use Committee (IACUC) of the National University of Singapore.

### Hippocampal slice preparation

Acute hippocampal slices were prepared using methods described previously [7, 73, 74]. In brief, the mice were anesthetized with CO_2_ and then decapitated. The whole brain was gently removed and kept in cold (4 °C) artificial cerebrospinal fluid (ACSF) saturated with carbogen (95% O_2_, 5% CO_2_; total consumption: 16 l/h). The ACSF used for field electrophysiological experiments was composed of the following (in mM): 124.0 NaCl, 3.7 KCl, 1.0 MgSO_4_.7 H_2_O, 2.5 CaCl_2_, 1.2 KH_2_PO_4_, 24.6 NaHCO_3_, and 10 D-glucose. Both right and left hippocampi were isolated and sliced into 400 μm-thick slices with a manual tissue chopper, at an angle of around 70° to the fimbria.

The slices were then transferred onto a nylon net and maintained at 32 °C in an interface chamber (Scientific System Design) with constant ACSF flow at a rate of 1 ml/min. Slices were incubated for at least 2–3 h before the start of field electrophysiological experiments. For all field electrophysiology recordings, one monopolar lacquer coated stainless steel electrode (5 MΩ; AM Systems) was positioned in the stratum radiatum of CA1 region for stimulating a synaptic input, S1. Another electrode was placed in the CA1 apical dendritic layer to record the field excitatory post synaptic potentials (fEPSP). The signals were amplified by a differential amplifier (Model 1700, AM systems), and digitized using a CED (Cambridge Electronic Design) and were monitored online.

The stimulation strength was determined by an input-output curve (stimulus intensity vs field EPSP slope) and was set to obtain a fEPSP slope of 40% of the maximum value. A custom made software, PWIN (Leibniz-Institute for Neurobiology, Magdeburg, Germany) was used to record and monitor signals online.

L-LTP was induced using a strong tetanus (STET) consisting of three stimulus trains of 100 pulses, 100 Hz; duration, 0.2 ms/polarity; intertrain interval, 10 min. A weak tetanization (WTET) protocol consisting of one 100 Hz train (21 biphasic constant-current pulses; pulse duration per half-wave, 0.2 ms) was used to induce E-LTP. L-LTD was induced using a strong low-frequency stimulation (SLFS) protocol consisting of 900 bursts (one burst consisted of three stimuli at 20 Hz, and the interburst interval was 1 s (i.e., f = 1 Hz; stimulus duration, 0.2 ms/half wave; total number of stimuli, 2700)). In experiments in which a weaker induction of LTD was investigated, a weak low-frequency stimulation protocol (WLFS) was used consisting of 900 pulses at a frequency of 1 Hz, an impulse duration of 0.2 ms/half wave, with 900 total stimuli. A stable baseline was recorded for ≥30 min before LTP/LTD induction; four 0.2 Hz biphasic constant-current pulses (0.1 ms per polarity) were used for baseline recording and testing at 1, 3, 5, 11, 15, 21, 25, and 30 min post-tetanus or 21, 25, and 30 min post-LFS and thereafter once every 5 min up to 180 min.

### Behavioural tagging

Associative memory was studied in Exon 33^−/−^ and WT mice using behavioural tagging (BT) paradigm as described [46, 74, 75]. A weak IA (Inhibitory Avoidance) training like a weak foot shock (0.3 mA, 2 s) induces only a short-term memory that lasts for a few hours. But this can be consolidated into long-term memory by novelty exploration (NE) consisting of 10 min of OF (open field), which induces protein synthesis, occurring 1 h before IA training [3, 27, 28]. For BT, mice were habituated to the room 12 h before the beginning of the experiments. The mice were grouped into two, and one group (control) was given IA training directly (Fig. 3A) and the other group was given a novelty exposure (NE) for 10 min, (Fig. 3B) and IA training was given 1 h after NE. The NE consisted of a plastic box with the dimensions 35 (width) × 35 (length) × 35 cm (height). The IA apparatus consisted of a 50 (width) × 25 (height) × 25 cm (length) Plexiglass box with a 5 (height) × 8 (width) × 25 cm (length) platform on the left end of a series of bars, that constitutes the floor of the box. During the IA training session, mice were placed on the platform in the corner of the box. When they stepped down, putting their four paws on the bars, they received a weak foot shock after which they were removed from the box and returned to their home cage. Memory was measured by comparing the step-down latency in the training session to that in test session and also between Exon 33^−/−^ and WT mice. The cut-off time for step-down latency was 4 min. A high step-down latency indicates that the animal has stronger memory. Memory was tested at three different time points: 1 h, 24 h, and 7 d after IA training session. Same animals were used for retesting at various time points in each group.

### Tube dominance test

Tube dominance test was performed to study social dominance by measuring aggression. Animals were handled daily for 5 days prior to the test. For habituation, all animals were allowed to freely explore the tube for 15 min one day before the test. The tube size was selected so that the mice could easily pass the tubes, but they were unable to pass over each other. Removable separators were placed at the entrances of the tube from each holding box, and at a central location. On the day of testing, one Exon 33^−/−^ mice and one WT mice were introduced into opposite ends of the tube, whereupon they will move to the centre of the tube, and then the central separator was removed to allow the dominance test to begin. The animals interacted in the tube and the more dominant animal would force its opponent out of the tube. Once one animal has all the paws out of the tube, it was declared the loser and the opponent was the winner. Each Exon 33^−/−^ mice was tested against each WT mice and the number of wins were recorded (Fig. 4A).

### Sociability and social novelty test

This test helps to investigate the general sociability of animals and their ability to recognize familiar versus novel animals. It is based on the fact that mice generally prefer to spend more time with other mice (sociability) and prefer to interact with a novel intruder than with a familiar mouse (social novelty). The apparatus for testing consisted of a plastic box with the dimensions 40 (width) × 40 (length) × 40 cm (height) with two grid cups placed at two diagonal corners. Animals were handled 5 days prior to the test. All animals were allowed to freely explore the apparatus with two empty grid cups for 15 min one day before the test. On the first day, the subject mouse encountered a never-before-met mouse (Mouse 1, a weight matched C57BL/6 J mice) in one grid cup and an empty cup in the box. This is the sociability test session, where social interaction parameters were recorded for 10 min: the time spend sniffing on each grid cup. Mice generally prefer to spend more time with another mouse than with an empty cup. On the second day, another weight matched never-before-met C57BL/6 J mice (Mouse 2) was introduced in one grid cup and the familiar one (Mouse 1) in the other grid cup. The grid cup for Mouse 1 was swapped between each test to prevent side preference of test animals. Normal mice tend to interact with a novel mouse more than a familiar mouse (social novelty) (Fig. 4C). To eliminate any possible observer bias, automated tracking with the TopScan system (CleverSys Inc.) was implemented.

#### Statistics

In field electrophysiological recordings, synaptic strength was determined as the slope of fEPSP (millivolts per millisecond). All values were taken as the mean of normalized fEPSP slope (percentage of baseline) ± SEM. For evaluating statistical significance, Wilcoxon signed-rank test (Wilcox test), was used to compare the mean normalized fEPSPs at specific time points with the baseline value at time point −15 min within the same group. To compare between different groups, Mann–Whitney U test (U-test), was applied. Nonparametric tests were selected because a normal distribution could not always be assumed with the sample size per series. Mann–Whitney U-test is non-parametric and not normally distributed, while Wilcoxon rank test approximates a normal distribution and is non-parametric. One-way ANOVA, with multiple comparisons were used for behavioural tagging experiments. Response variables are normally distributed for one-way ANOVA. For social interaction tests, two-way ANOVA with multiple comparison were used. Differences were considered as statistically significant when *P* < 0.05.

## Supplementary information

Enhanced LTP in Exon 33-/- is NMDA-receptor independent

Gross phenotypic characteristics of Exon 33-/- mice

Methods
