# Supplementary material for: Enhanced long-term potentiation and impaired learning in mice lacking alternative exon 33 of CaV1.2 calcium channel
Source: Transl Psychiatry. 2022 Jan 10;12:1. doi: 10.1038/s41398-021-01683-2 (PMC8748671; doi:10.1038/s41398-021-01683-2)
Supplement: Supplementary file 1 — Enhanced LTP in Exon 33-/- is NMDA-receptor independent [file 41398_2021_1683_MOESM1_ESM.docx]

**Supplementary Figures and Information**

**S.Fig. 1 legend**

Enhanced LTP in Exon 33^-/-^ is NMDA-receptor independent: After recording a stable baseline for 30 min, AP5 (50 µM), an NMDA-receptor antagonist was bath applied for 60 min. 30 min after AP5 application STET was delivered to S1 which resulted in a statistically significant potentiation that maintained for 180 min (*P>0.05)*. Three solid arrows represent the time of induction of L-LTP by STET for the induction of late-LTP. The rectangular box represents the time point of application of AP5. Scale bars: vertical, 2 mV; horizontal, 3 ms. STET-strong tetanisation, WTET-weak tetanisation.

**S. Fig. 2 legend**

Gross phenotypic characteristics of Exon 33^-/-^ mice: A.Optokinetic drum vision test. Two different width black and white strips (0.5 cm and 1 cm) were used with rotating in clock wise and counter-clock wise. The optokinetic reflex of the tested animals is observed as its gaze following the strips. Trails were scored as 1 for success and 0 for failure. Both WT and Exon 33^-/-^ mice showed normal optokinetic reflex (WT n=3, Exon 33^-/-^ n=4). B.Acoustic startle response Test. Startle responses of animals were recorded with different sound stimulation. Both WT and Exon 33^-/-^ mice showed increasing startle response with raising intensity of sound stimulus (WT n=3, Exon 33^-/-^ n=4). C.Body weight of WT and Exon 33^-/-^ mice at 3 month age. There is no difference in body weight between WT and Exon 33^-/-^ mice (WT n=7, Exon 33^-/-^ n=6).D. Open field test. The locomotor activity was tested by using open field test. Velocity of animals during the test was measured by TopScan software. There is no difference in locomotor activity between WT and Exon 33^-/-^ mice (WT n=6, Exon 33^-/-^ n=6).

**Supplementary information**

Optokinetic drum vision test.

Animals were placed on the platform in the center of drum for 3 min. Two different width black and white strips (0.5 cm and 1 cm) were used with rotating in clock wise and counter-clock wise. The optokinetic reflex of the tested animals is observed as its gaze following the strips. Trails were scored as 1 for successful observation of gaze behaviour 3 times during the test and 0 for animals who failed to do so.

Acoustic startle response test.

Acoustic startle responses of animals were carried out using SR-LAB startle chambers (San Diego Instruments, San Diego, CA). The animals were placed inside the plexiglas cylinder to accliminate for 5 min before playing sound stimulus. Sound stimulus with intensity of 0, 70, 75,80,85,90,100,110 and 120 dB were played in random orders for 5 times. The startle response of animals were measured by the sensors that convert small movements to voltage. The magnitude of the change in voltage represented the startle response of the tested animals. Averge change in voltage in response to the sound stimulus with same intensity was calculated from 5 repeated trails.

Open field test.

Locomotor activity for individual animals was monitored in sqaure open field in a plexiglass cage with the dimensions 40 (width) × 40 (length) × 40 cm (height). The measurement was performed under approcximal 180 lux illumination. The animals were placed in the apparatus for 30 min to obtain locomotor activity. Automated tracking of animals were implemented by TopScan system (CleverSys Inc.). Locomotion was measured by the velocity of mice during the test.
