# Supplementary figures and images for: Enhanced long-term potentiation and impaired learning in mice lacking alternative exon 33 of CaV1.2 calcium channel
Source: Transl Psychiatry. 2022 Jan 10;12:1. doi: 10.1038/s41398-021-01683-2 (PMC8748671; doi:10.1038/s41398-021-01683-2)

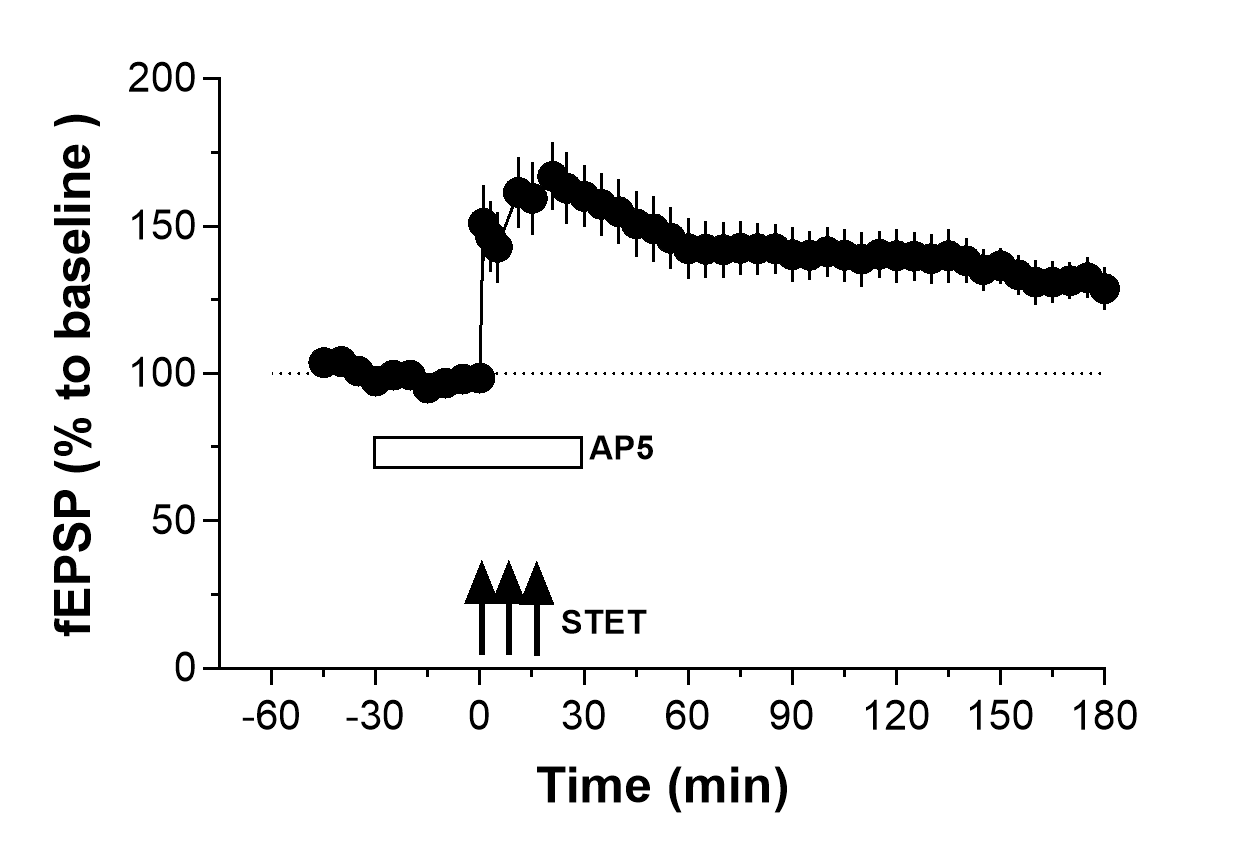

Supplement: Supplementary file 2 — Gross phenotypic characteristics of Exon 33-/- mice [file 41398_2021_1683_MOESM2_ESM.tif]

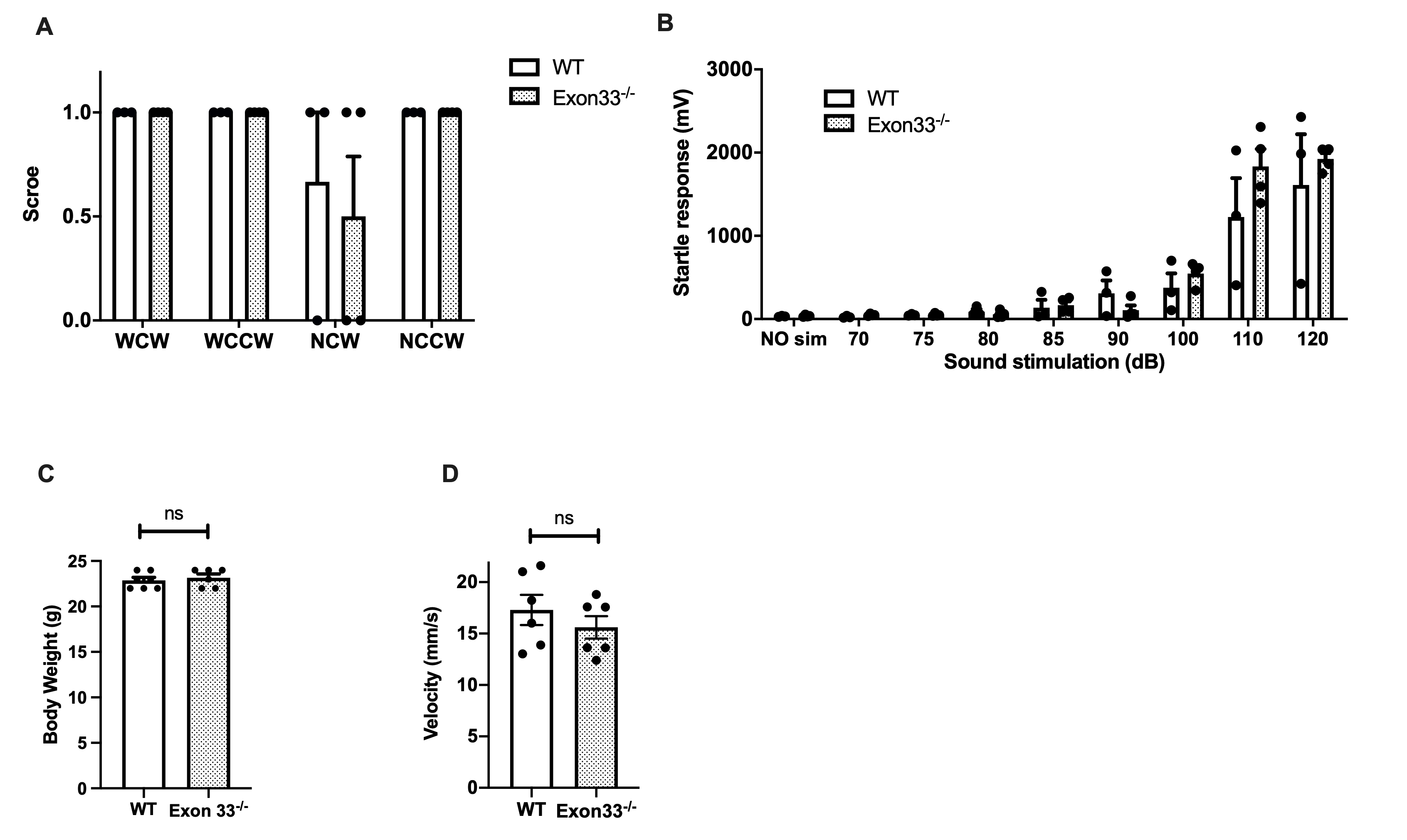

Supplement: Supplementary file 3 — Methods [file 41398_2021_1683_MOESM3_ESM.tif]
